# Dietary patterns and colorectal cancer: results from a Canadian population-based study

**DOI:** 10.1186/1475-2891-14-8

**Published:** 2015-01-15

**Authors:** Zhi Chen, Peizhong Peter Wang, Jennifer Woodrow, Yun Zhu, Barbara Roebothan, John R Mclaughlin, Patrick S Parfrey

**Affiliations:** Division of Community Health and Humanities, Faculty of Medicine, Memorial University of Newfoundland, St. John’s, Newfoundland and Labrador, A1B 3V6 Canada; Clinical Epidemiology Unit, Faculty of Medicine, Memorial University of Newfoundland, St John’s, NL Canada; Samuel Lunenfeld Research Institute, Mount Sinai Hospital, Toronto, ON Canada

**Keywords:** Exploratory factor analysis, Colorectal cancer, Case–control study, Dietary pattern, Newfoundland and Labrador population

## Abstract

**Background:**

The relationship between major dietary patterns and colorectal cancer (CRC) in other populations largely remains consistent across studies. The objective of the present study is to assess if dietary patterns are associated with the risk of CRC in the population of Newfoundland and Labrador (NL).

**Methods:**

Data from a population based case–control study in the province of NL were analyzed, including 506 CRC patients (306 men and 200 women) and 673 controls (400 men and 273 women), aged 20–74 years. Dietary habits were assessed by a 169-item food frequency questionnaire (FFQ). Logistic regression analyses were performed to investigate the association between dietary patterns and the CRC risk.

**Results:**

Three major dietary patterns were derived using factor analysis, namely a Meat-diet pattern, a Plant-based diet pattern and a Sugary-diet pattern. In combination the three dietary patterns explained 74% of the total variance in food intake. Results suggest that the Meat-diet and the Sugary-diet increased the risk of CRC with corresponding odds ratios (ORs) of 1.84 (95% CI: 1.19-2.86) and 2.26 (95% CI: 1.39-3.66) for people in the highest intake quintile compared to those in the lowest. Whereas plant-based diet pattern decreases the risk of CRC with a corresponding OR of 0.55 (95% CI: 0.35-0.87). Even though odds ratios (ORs) were not always statistically significant, largely similar associations across three cancer sites were found: the proximal colon, the distal colon, and the rectum.

**Conclusion:**

The finding that Meat-diet/Sugary-diet patterns increased and Plant-based diet pattern decreased the risk of CRC would guide the promotion of healthy eating for primary prevention of CRC in this population.

## Introduction

Studies on diet and chronic diseases suggest that lifestyle factors, especially dietary habits and physical activities, play major roles in causing or preventing colorectal cancer (CRC) [1, 2]. There has been an increased interest in associations between dietary factors and CRC for a while; several articles on this subject have been published by our research group, a large and diverse multidisciplinary team of more than 40 researchers from Newfoundland and Labrador (NL) and Ontario (ON) [3–6]. Most previous researchers have focused on the effects of a single food or nutrient; for example, Sun et al. [3, 4] reported that selected micronutrients (e.g., calcium, vitamin D, vitamin C, folate) are associated with a lower risk of incident CRC, while diets high in macronutrients (i.e., protein, fiber, and carbohydrate) may reduce the risk of the disease. However, studies of single food items or groups in relation to CRC may not be valid because they assume that each single food or nutrient has an isolated effect [7–9]. The dietary pattern approach, which has been increasingly used in nutritional epidemiology, could capture and assess the overall dietary experience through considering simultaneous effects of dietary exposures potentially interacting with each other [10]. Zhu et al. [6] explored the effects of dietary patterns on CRC patients’ survival and suggested that the processed meat pattern, which is characterized by higher intake of red meat, cured/processed meat, fish and processed fish, is associated with a decreased disease-free survival after CRC diagnosis.

Even though considerable differences exist between population characteristics, study designs, and the methodologies used for conducting dietary pattern analysis, the results pertaining to the relationship between diet and CRC from previous studies applying this approach were nearly consistent [11, 12]. Generally, the patterns that were labelled as “healthy” or “prudent”, mainly characterized by higher consumption of fruits, vegetables, and grains, and lower consumption of sweets, red meat, and processed meat, were associated with a lower risk of CRC. Conversely, diets defined as “western”, which indicate higher intakes of meat, highly processed food, potatoes, and refined carbohydrates, as well as lower intakes of greens and dietary fibre, have been associated with an increased CRC risk [12–15].

However, due to the effects of individual dietary habits, geographic factors and cultural differences, the dietary pattern approach is population-dependent, which may limit the external validity of existing findings [10]. Therefore, in order to translate this knowledge into dietary recommendation for different populations, population-specific studies using this methodology are needed. The present study aims to identify the association between dietary patterns and CRC in a Canadian population, from the province of NL.

## Methods

### Study design

A case–control study was conducted for the investigation of dietary patterns and CRC in the NL population. This study uses existing data that was collected by the Newfoundland Familial Colorectal Cancer Registry (NFCCR).

### Study participants

A detailed description of study participants can be found elsewhere [3, 5, 16, 17]. Briefly, eligible cases were newly diagnosed CRC patients identified from the NFCCR during 1999–2003, between the ages of 20–74 years. Incident CRC diagnosis was identified through International Classification of Diseases 9th revision codes (ICD-9 codes): 153.0-153.9, 154.1-154.3 and 154.8; or ICD-10 codes: 18.0-18.7, 19.9, 20.9. Controls were selected from the NL population through random-digit dialing using telephone numbers provided by Aliant (a local telephone company in NL). They were frequency-matched with cases, also aged 20–74 years, by sex and age on 5-year strata [16, 17]. Both cases and controls were residents of NL at time of diagnosis or interview.

A written consent form, personal history questionnaire (PHQ), and food frequency questionnaire (FFQ) were sent to each case and control who agreed to participant in this survey. Based on PHQ returning, the analytical sample sizes for the present study were 703 cases and 717 controls. However, only those participants who completed both PHQ and FFQ were entered into final analysis. Hence, the total sample size is 1204 (518 cases and 686 controls) [17].

### Data collection

Dietary intake data was gathered using a modified FFQ, based on the validated Hawaii FFQ, that was adapted to include foods particular to NL (e.g., cloudberries, game, and pickled/smoked fish). The modified version of the FFQ has been validated by our team and was widely used in the province of NL [18]. Diet assessment in this FFQ was carried out one to two years prior to diagnosis or interview. Herein, interview indicates this survey on PHQ and FFQ. The 169 food items listed in the FFQ were categorized into nine major groups: beverages; dairy products; mixed dishes; vegetables; meat and fish; cereals and grains; fruits; desserts and sweets; and miscellaneous. Participants were required to recall the frequency of food intake and their usual portion size from the choices “smaller”, “average”, and “larger”, based on food photographs indicating examples of portion sizes. A “smaller” size means 75% of an “average” size while a “larger” size is defined as 125% of an “average” size. Total energy intakes were calculated based on the composition values from the 2005 Canadian Nutrient file, by multiplying the frequency of each food item by the calories contained in each portion [3].

The PHQ was applied to gather socio-demographic information, such as age, sex, date of birth, marital status, educational attainment, medical history, bowel screening history, medication use, physical activity, reproductive factors (females only), alcohol and tobacco use.

For this analysis, we excluded those who did not provide sufficient dietary information at baseline, or failed to provide information on potential risk factors at baseline. In addition, those who reported energy intakes outside the range 500–5000 calories/day were excluded [19]. After the exclusion, 1179 participants (506 cases and 673 controls), who completed both the PHQ and FFQ, remained for further analysis.

### Statistical analysis

The 169 food items in the FFQ were divided into 39 food groups based on the roles of food in diet and nutritional characteristics. Several foods that could not be appropriately combined with others were defined as their own groups; for example, eggs, jams, beer, and fruit pies. Exploratory factor analysis was used to identify major dietary patterns for both cases and controls recruited from the NL population, based on the 39 predefined food groups. These factors were rotated by a varimax rotation (orthogonal) procedure for greater interpretability, uncorrelated components and greatest amount of variance explained. Factors were retained according to the following criteria: factor eigenvalue greater than 1.15; the break point of the scree plot; the proportion of variance explained; and factor interpretability [20]. Patterns were labelled based on food groups with absolute rotated factor loadings equal to or greater than 0.35. A factor score calculated for each dietary pattern (factor) by loading matrix was assigned to each participant, indicating the extent to which their diet corresponded to that pattern. In other words, an individual with a higher factor score has a stronger adherence to that pattern.

Two unconditional logistic models were used to calculate the odds ratios (OR) and the corresponding 95% confidence intervals (CI) that were used to interpret the associations between dietary patterns and CRC risk. The original models were adjusted only for age and total energy intake. The multivariate regression analyses were used to further adjust for additional confounding factors. They included sex, body mass index (BMI), marital status, educational attainment, household income status; use of alcohol, tobacco, non-steroidal anti-inflammatory drugs (NSAIDs); family history of CRC; history of polyps, diabetes, colon screening procedures, high cholesterol, Crohns disease or colitis; multivitamin supplement use; and physical activities. Generally, potential confounding factors were selected into models according to the results of the literature review or biological plausibility. Additionally, in order for a factor to be selected there must be a 10% or more change in the regression coefficient of the primary predictors after addition of the factors and the model must have a p-value <0.05 when the covariate is entered. Factor scores assigned to each participant were categorized into quintiles and entered into each model as independent variables, with the lowest quintile as the reference group; the outcome variable is the status of each participant (CRC patient or control) [4]. P values for trend were calculated by Mantel-Haeszel Chi-Square Test to assess dose–response relationships.

Statistical analyses were carried out using Statistical Analysis System (SAS, version 9.2) software. All statistical tests were two-sided, and p-values < 0.05 were considered statistically significant.

#### Ethical consideration

This research was approved by the Health Research Ethics Board (HREB) at Memorial University of Newfoundland. (Reference number 14.098).

## Results

The socio-demographic, lifestyle and medical characteristics of the 506 cases and 673 controls are shown in Table 1. Due to frequency-matched design, the gender distribution is similar in cases and controls (p > 0.05). Cases (62.5 ± 9.2) are significantly older than controls (60.5 ± 9.5) (p = 0.0003). Difference in mean of total energy intake between the case (2444.3 ± 890.9) and control (2259.2 ± 784.6) group is significant (p = 0.0003). Compared to controls, cases tended to be less educated; more obese (BMI ≥ 30); either physically inactive (0 ~ 7.4 hours/week) or extremely physically active (>53.0 hours/week); more likely to have a history of polyp, diabetes and smoking; and less colon screening procedure and NSAIDs use (p < 0.05). No significant difference was found in other baseline factors between the two groups.

**Table 1:** **Characteristics of case and control groups**

Variables	Cases (n = 506) n (%)	Controls (n = 673) n (%)	p-value
**Gender**			
Male	306 (60.47%)	400 (59.44%)	
Female	200 (39.53%)	273 (40.56%)	
**Level of education**			<0.0001
Less than 11 years	246 (48.71%)	210 (31.44%)	
High school graduate	75 (14.85%)	103 (15.42%)	
Vocational or technical school/college	141 (27.92%)	247 (36.98%)	
Bachelor/graduate degree	43 (8.51%)	108 (16.17%)	
**Marital status**			
Single/never married	30 (5.93%)	22 (3.27%)	
Separated, divorced or widowed	80 (15.81%)	100 (14.86%)	
Currently married or living as married	396 (78.26%)	548 (81.43%)	
**Household income (per year)**			<0.0001
Less than $12,000	40 (8.59%)	43 (7.20%)	
$12,000-$19,999	181 (40.49%)	185 (30.99%)	
$30,000-$49,000	132 (29.53%)	163 (27.30%)	
More than $50,000	94 (21.03%)	206 (34.51%)	
**Body mass index (kg/m** ^**2**^ **)**			0.004
15-18.5	7 (1.42%)	2 (0.30%)	
18.5-25	135 (27.38%)	205 (31.20%)	
25-30	206 (41.78%)	306 (46.58%)	
More than 30	145 (29.41%)	144 (21.96%)	
**Physical activity (hours/week)**			0.037
0-7.4	140 (27.72%)	166 (25.08%)	
7.4-22.4	90 (17.82%)	148 (22.36%)	
22.4-53.0	96 (19.01%)	151 (22.81%)	
More than 53.0	179 (35.45%)	197 (29.76%)	
**Family history of colorectal cancer**	54 (10.67%)	55 (8.18%)	
**Polyp**	240 (48.29%)	85 (12.98%)	<0.0001
**Diabetes**	107 (21.15%)	89 (13.40%)	0.0004
**High cholesterol/triglycerides**	169 (33.47%)	258 (38.39%)	
**Crohns disease or colitis**	12 (2.42%)	13 (2.00%)	
**Any colon screening procedure**	65 (12.85%)	145 (21.55%)	0.0001
**Smoking status**	367 (72.53%)	422 (62.70%)	0.0004
**Alcohol use**	301 (59.49%)	379 (56.32%)	
**Multivitamin use**	101 (20.08%)	146 (21.82%)	
**Non-steroidal anti-inflammatory drug use**	167 (33.07%)	260 (38.75%)	0.045

Significant level at 0.05; non-significant p-values are not shown.

Three major dietary patterns were derived using exploratory factor analysis and factor labelling; the three patterns are shown in Table 2. These three dietary patterns explained 74% of variance. A predefined food group was considered as being loaded on a specific pattern when its absolute factor loading was ≥ 0.35. The first pattern was defined as Meat-diet pattern, which is characterized by high loadings for red meat, cured/processed red meat, fish, and processed fish. The second pattern, which loaded heavily on root vegetables, tomato sauce, total cereals and grains, berries, dried fruits, other fruits, other green vegetables, and other vegetables, was labelled as Plant-based diet pattern. The final pattern was named Sugary-diet pattern because it has high loadings of pies, tarts, desserts, and sweets.

**Table 2:** **Factor loadings and explained variances (VAR) for the three major dietary patterns identified in an adult NL population, using a common factor analysis** ^**a**^

Food groups	Dietary pattern		
	Meat-diet	Plant-based diet	Sugary-diet
Milk		0.19	
Yogurt		0.31	
Coffee	0.18		
Tea			0.17
Sugar		-0.19	0.20
Soft drinks	0.19		
Egg	0.21		0.16
Cheese	0.15	0.21	
Mixed dishes	0.31	0.17	0.24
Red meat	**0.68**		0.18
Game	0.24		
Cured /processed red meat	**0.72**		0.21
Cured /processed meat	**0.93**		
Poultry	0.22	0.27	
Fish	**0.59**	0.31	-0.21
Processed fish	**0.51**	0.24	
Fruit juice		0.24	0.24
Other fruits		**0.59**	
Root vegetables	0.28		0.15
Cruciferous vegetables		**0.54**	
Other greens		**0.60**	-0.22
Beans, peas	0.15	0.25	
Tomato sauce		**0.50**	
Other vegetables	0.22	**0.54**	
Total cereals and grains	0.23	**0.38**	0.28
Whole grains		0.33	
Desserts and sweets	0.31		**0.63**
Vegetable juice		0.17	
Beer	0.19		
White wine			
Red wine			
Liquor	0.15		
Citrus		0.34	
Berries		**0.45**	
Dried fruit		**0.39**	
Canned fruit		0.20	0.24
Pies, tarts	0.15		**0.54**
Jam, jelly			0.26
Pickled vegetables	0.15	0.26	0.14
Proportion of VAR explained (%)	40%	23%	11%
Cumulative VAR explained (%)	40%	63%	74%

^a^Absolute values less than 0.15 were not listed and those above 0.35 indicated in bold to visually emphasize strength of association.

Table 3 presents the ORs and their 95% CIs for CRC by the quintiles of factor scores for each dietary pattern. After adjusting for potential covariates, the higher risk of CRC is associated with the Meat-diet pattern (the highest vs. the lowest quintiles: OR = 1.84; 95% CI = 1.19 ~ 2.86), and the Sugary-diet pattern (the highest vs. the lowest quintiles: OR = 2.26; 95% CI = 1.39 ~ 3.66). The factor scores for the Plant-based diet pattern are reversely related to the risk of CRC (the highest vs. the lowest quintiles: OR = 0.55; 95% CI = 0.35 ~ 0.87).

**Table 3:** **Odds ratios and 95% CI of colorectal cancer according to the three major dietary patterns in a NL population**

Dietary pattern	Quintiles									
	Q1	Q2		Q3		Q4		Q5		P for trend
		OR	95% CI	OR	95% CI	OR	95% CI	OR	95% CI	
Meat-diet pattern										
^†^original	1.00	1.52	**1.04, 2.23**	1.37	0.94, 2.03	1.86	**1.27, 2.72**	2.14	**1.41, 3.24**	0.0002
^‡^multivariate	1.00	1.44	0.91, 2.30	1.32	0.88, 1.97	1.64	**1.09, 2.46**	1.84	**1.19, 2.86**	0.6442
Plant-based diet pattern										
^†^original	1.00	1.04	0.72, 1.51	0.73	0.50, 1.06	0.69	**0.47, 0.99**	0.46	**0.31, 0.69**	<0.0001
^‡^multivariate	1.00	1.09	0.74, 1.60	0.78	0.53, 1.17	0.77	0.51, 1.16	0.55	**0.35, 0.87**	0.3065
Sugary-diet pattern										
^†^original	1.00	1.69	**1.15, 2.48**	1.55	**1.06, 2.27**	2.20	**1.48, 3.27**	2.51	**1.64, 3.84**	<0.0001
^‡^multivariate	1.00	1.47	0.96, 2.26	1.46	0.94, 2,25	1.92	**1.23, 3.01**	2.26	**1.39, 3.66**	0.4024

^†^Adjusted for age and total energy intake;

^‡^Adjusted for: sex, body mass index, marital status, education attainment, household income status, use of alcohol/tobacco/non-steroid anti-inflammatory drug (NSAID), family history of CRC, history of diabetes/colon screening procedure/ high cholesterol, reported hormone replacement therapy (females only), multivitamin supplements, and physical activities;

Significant 95% CI are in bold;

P for trend was calculated by Mantel-Haenszel Chi-Square Test.

In order to further clarify the effects of the three dietary patterns, logistic regression models were fitted by proximal colon cancer, distal colon cancer and rectal cancer, respectively (Table 4). After adjusting for potential confounders, no significant effects of the Meat-diet and Plant-based diet pattern on proximal colon cancer were detected. However, the Sugary-diet pattern is associated with higher risk of proximal colon pattern (the highest vs. the lowest quintiles: OR = 2.90; 95% CI = 1.54 ~ 5.45). As for distal colon cancer, higher risk is significantly associated with the Meat-diet pattern (the highest vs. the lowest quintiles: OR = 2.29; 95% CI = 1.16 ~ 4.53) and the Sugary-diet pattern (the highest vs. the lowest quintiles: OR = 2.40; 95% CI = 1.20 ~ 4.81), and non-significantly inversely related to the Plant-based diet pattern (the highest vs. the lowest quintiles: OR = 0.72; 95% CI = 0.35 ~ 1.45). Additionally, the Meat-diet (the highest vs. the lowest quintiles: OR = 2.01; 95% CI = 1.06 ~ 3.80) and Plant-based diet pattern (the highest vs. the lowest quintiles: OR = 2.01; 95% CI = 1.01 ~ 4.00) are significantly associated with higher risk of rectum cancer. However, the Plant-based diet pattern is inversely related to the risk of rectum cancer (the highest vs. the lowest quintiles: OR = 0.46; 95% CI = 0.23 ~ 0.90).

**Table 4:** **Odds ratios and 95% CI for cancer of the proximal colon, distal colon and rectum according to the three major dietary patterns in a NL population**

Dietary pattern	Quintiles									
	Q1	Q2		Q3		Q4		Q5		P for trend
		OR	95% CI	OR	95% CI	OR	95% CI	OR	95% CI	
**Proximal colon cancer**										
Meat-diet pattern										
^†^original	1.00	1.42	0.84, 2.41	1.43	0.85, 2.40	1.57	0.93, 2.65	1.30	0.72, 2.33	0.2501
^‡^multivariate	1.00	1.45	0.85, 2.50	1.50	0.88, 2.57	1.61	0.94, 2.78	1.34	0.72, 2.47	0.3182
Plant-based diet pattern										
^†^original	1.00	1.34	0.80, 2.24	0.91	0.53, 1.55	0.83	0.48, 1.43	0.70	0.40, 1.24	0.1287
^‡^multivariate	1.00	1.31	0.77, 2.24	0.88	0.48, 1.45	0.78	0.44, 1.38	0.64	0.34, 1.19	0.4062
Sugary-diet pattern										
^†^original	1.00	1.52	0.86, 2.69	1.72	0.99, 3.00	2.23	**1.27, 3.93**	2.75	**1.51, 5.03**	0.0009
^‡^multivariate	1.00	1.70	0.95, 3.04	1.69	0.96, 2.99	2.39	**1.32, 4.33**	2.90	**1.54, 5.45**	0.1144
**Distal colon cancer**										
Meat-diet pattern										
^†^original	1.00	1.63	0.87, 3.05	1.55	0.83, 2.90	1.68	0.89, 3.17	2.87	**1.51, 5.44**	0.0035
^‡^multivariate	1.00	1.55	0.81, 2.97	1.33	0.69, 2.55	1.43	0.74, 2.80	2.29	**1.16, 4.53**	0.0035
Plant-based diet pattern										
^†^original	1.00	1.14	0.64, 2.03	0.80	0.44, 1.45	0.82	0.45, 1.47	0.54	0.28, 1.02	0.0534
^‡^multivariate	1.00	1.27	0.69, 2.32	0.90	0.48, 1.68	0.99	0.53, 1.87	0.72	0.35, 1.45	0.6609
Sugary-diet pattern										
^†^original	1.00	1.63	0.86, 3.09	1.52	0.81, 2.88	2.28	**1.21, 4.28**	2.78	**1.44, 5.40**	0.0024
‡multivariate	1.00	1.56	0.81, 3.01	1.55	0.80, 3.01	2.14	**1.11, 4.14**	2.40	**1.20, 4.81**	0.0797
**Rectum cancer**										
Meat-diet pattern										
^†^original	1.00	1.65	0.92, 2.94	1.18	0.65, 2.17	2.39	**1.37, 4.18**	2.78	**1.54, 5.03**	0.0005
^‡^multivariate	1.00	1.61	0.88, 2.97	1.02	0.54, 1.93	2.11	**1.16, 3.84**	2.01	**1.06, 3.80**	0.0183
Plant-based diet pattern										
^†^original	1.00	1.14	0.49, 1.34	0.57	**0.34, 0.96**	0.55	**0.33, 0.92**	0.27	**0.15, 0.49**	<0.0001
^‡^multivariate	1.00	0.93	0.54, 1.59	0.72	0.41, 1.27	0.85	0.48, 1.51	0.46	**0.23, 0.90**	0.1316
Sugary-diet pattern										
^†^original	1.00	1.90	**1.09, 3.31**	1.39	0.78, 2.47	2.06	**1.17, 3.64**	1.99	**1.08, 3.69**	0.0304
^‡^multivariate	1.00	1.97	**1.08, 3.58**	1.29	0.69, 2.41	1.96	**1.04, 3.69**	2.01	**1.01, 4.00**	0.6538

^†^Adjusted for age and total energy intake;

^‡^Adjusted for: sex, body mass index, marital status, education attainment, household income status, use of alcohol/tobacco/non-steroid anti-inflammatory drug (NSAID), family history of CRC, history of diabetes/colon screening procedure/ high cholesterol, reported hormone replacement therapy (females only), multivitamin supplements, and physical activities;

Significant 95% CI are in bold;

P for trend was calculated by Mantel-Haenszel Chi-Square Test.

## Discussion

Three major dietary patterns were derived for the NL population, including the Meat-diet, Plant-based diet and Sugary-diet pattern, which are highly consistent with another project conducted by our team for exploring the association between dietary pattern and CRC survival [6]. This case–control study further suggested that the Plant-based diet pattern conferred a protective effect against CRC, while the Meat-diet pattern and the Sugary-diet pattern were associated with a greater risk of CRC. After analyzing by proximal colon cancer, distal colon cancer, and rectum cancer, even though ORs were not always statistically significant, similar associations were found.

Our findings regarding less healthy patterns, such as the Meat-diet pattern and the Sugary-diet pattern, are largely in an agreement with those of other comparable studies that used factor analysis to derive dietary patterns. A study conducted in US population [21] indicates that the Western pattern characterized by a high consumption of sweets and desserts, red and processed meats, refined grains, and French fries was associated with increased CRC risk. Slattery et al. [22] conducted a factor analysis in a multicenter US population and identified a Western pattern characterized by higher intakes of red meat, processed meat, and sugar-containing food, that is related to an increased risk of colon cancer in both genders. From a case–control study conducted in Western New York, Randall et al. [23] identified a Traditional pattern of meat and baked goods that was associated with a higher risk of colon cancer. Furthermore, the overall conclusions from two recent systematic reviews addressing this topic are compatible with our results. In one of the reviews, the less healthy pattern with higher intakes of red and processed meat, potatoes and refined carbohydrates was associated with a higher risk of CRC [11]. Another review supposed that the self-labelling diet as “Western” was related to an increased risk of CRC with ORs ranging from 1.18 to 11.7 [12].

A healthier pattern with vegetables, fruits and other healthy foods which has been generally considered protective against the incidence and development of CRC was identified from previous studies [22, 24]. According to Fung et al’s [21] study in US population, a prudent pattern of vegetables, fruits, legumes, fish, poultry and whole grains was reported to be inversely, but not significantly, associated with colon cancer. Another US population-based case–control study reported a similar and significant association between a prudent pattern, which is characterized by higher intakes of vegetables and fruits, and a reduced risk of colon cancer in both genders [22]. Randall et al. [23] suggested a significant association between the healthier pattern (that is, salad vegetables) and a decreased risk of colon cancer in women, but insignificant one in men. Additionally, other studies conducted in different populations, including Asian people, have also suggested that a diet with higher intakes of fruits, vegetables, cereals, legumes and low fat dairy products would be protective against CRC [24–26].

We hypothesized that the Plant-based diet pattern would be associated with a reduced risk of CRC, but there was no strong significant evidence of this in this NL population, after analyzing by proximal colon cancer, distal colon cancer, and rectum cancer. Through fitting multivariable logistic regression models, only a significantly inverse association between the Plant-based diet pattern and rectum cancer was found. However, this healthier pattern is non-significantly inversely related to the risk of proximal and distal colon cancer. Even though the direction of this association is similar to the findings from other studies, it is not significant [22–26].

High consumption of red meat, processed meat, sweets and processed sugar, which are typical characteristics of the Meat-diet and Sugary-diet patterns, might determine these patterns’ relationship with CRC. The causal mechanism could involve overweight and obesity, which previous studies have found to be important risk factors for CRC [27–29]. From a study conducted among Hispanic women, an association between an animal protein pattern and a greater than three-fold increased risk of obesity was reported [30]. Murtaugh et al. [31] conducted a cross-sectional study in Iranian population and suggested that a western pattern with a higher intake of sweets and desserts, and red and processed meat was positively associated with obesity. Another possible mechanism is that heme, sodium nitrate, nitrite and N-nitroso compounds, which were found in lots of red meat and processed meat, have been associated with higher CRC risk [32–35].

In this study, fruits, vegetables and whole grains were loaded to the factor labelled as the Plant-based diet pattern. One possible mechanism of their protective effects against CRC is that they are good sources of vitamins A, C, and E, fibers, minerals, selenium, and carotenoids [36, 37]. These nutrients could have the effect of binding and diluting carcinogens as well as an anti-oxidant effect to change the physical environment in colonic flora, thereby affecting the incidence and development of CRC [36, 38].

Based on existing literature, this appears to be the first study that focuses on the relationships between dietary pattern and CRC in a Canadian population and provides updated information that may be applied to guide public health action for primary prevention of CRC. This study has a number of strengths. First of all, this study was conducted on a large sample which increases the likelihood of observing associations that would be impossible to detect in smaller studies. Secondly, instead of single nutrient/food approach, we used exploratory factor analysis to derive new non-correlated variables to explain the variation in dietary habits, thereby allowing us to obtain a more comprehensive and accurate picture of dietary exposures in this population. Thirdly, the FFQ used for this study, modified from the Hawaii FFQ, has been adapted to include regional foods consumed in NL and has been validated by our team [18]. When exploring the relationships of dietary patterns and CRC risk, multivariate logistic regression models that controlled for a wide range of potential confounding factors were fitted. Finally, two logistic regression models were adjusted for total energy intake. Between-person variation generated by over-reporting or under-reporting of food intakes were reduced by this adjustment [39].

The methodological limitations of case–control studies in general, and specifically shortcomings on the design and data analysis choices of this study, which may have influenced the observed associations, should be discussed. First of all, selection and recall bias are possible as in most case–control studies. Because exposure information was collected after diagnosis, differential recall between cases and controls could bias the results. Specifically, cases may recall their diets differently than controls because of their disease status [40]. In addition, controls who agreed to join this study may have done so because of an interest in health and may therefore have healthier dietary and physical activity habits. The differences in dietary pattern between the selected controls and cases may be larger than with truly comparable controls. Second, related to the design, cases and controls had similar sex distribution but not well-comparable age groups. Third, the factor retained, self-labelling and interpretation of the dietary patterns is somewhat arbitrary; however, the patterns derived for this study population have emerged repeatedly across studies that applied factor analysis or cluster analysis to determine dietary patterns in different populations [21–26].

## Conclusion

The present study demonstrated that diets that are characterized by a high consumption of red meat, processed meat, fish and processed fish (labelled as the Meat-diet pattern) or with a high consumption of fruit pies, tarts, desserts and sweets (labelled as the Sugary-diet pattern) are associated with an increased risk of CRC in a Canadian population. However, the Plant-based diet pattern of fruits, vegetables and whole grains has a protective effect against CRC. In addition, the diet-disease relationships investigated here could be used to develop targeted interventions aimed at promoting healthy eating habits, with the goal of preventing CRC in Canada, and particularly in the NL population.

## Abbreviations

BMI: Body mass index
CI: Confidence interval
CRC: Colorectal cancer
FFQ: Food-frequency Questionnaire
NFCCR: Newfoundland familial colorectal cancer registry
NL: Newfoundland and Labrador
NSAIDs: Non-steroidal anti-inflammatory drugs
ON: Ontario
OR: Odds ratio
PHQ: Personal history questionnaire
SAS: Statistical analysis system.
